# Supplementary material for: Reconciling contrasting views on economic complexity
Source: Nat Commun. 2020 Jul 3;11:3352. doi: 10.1038/s41467-020-16992-1 (PMC7335174; doi:10.1038/s41467-020-16992-1)
Supplement: Supplementary file 1 — Supplementary Information [file 41467_2020_16992_MOESM1_ESM.pdf]

**Supplementary Information**

# Reconciling contrasting views on economic complexity

SCIARRA ET AL.

# CONTENTS

**Supplementary Figures** .... 2
Supplementary Figure 1 .... 2
Supplementary Figure 2 .... 3
Supplementary Figure 3 .... 4
Supplementary Figure 4 .... 7
Supplementary Figure 5 .... 8
Supplementary Figure 6 .... 9
Supplementary Figure 7 .... 11
Supplementary Figure 8 .... 10
Supplementary Figure 10 .... 6
Supplementary Figure 11 .... 12
**Supplementary Tables** .... 13
Supplementary Table 1 .... 13
**Supplementary Notes** .... 16
Supplementary Note 1: Implementation of the algorithms .... 16
Supplementary Note 2: The knee-like shape .... 16
Supplementary Note 3: The regimes of growth .... 18
Supplementary Note 4: The complexity of products .... 16
Supplementary Note 5: Ecological networks .... 19
**Supplementary References** .... 20

# SUPPLEMENTARY FIGURES

## SUPPLEMENTARY FIGURE 1

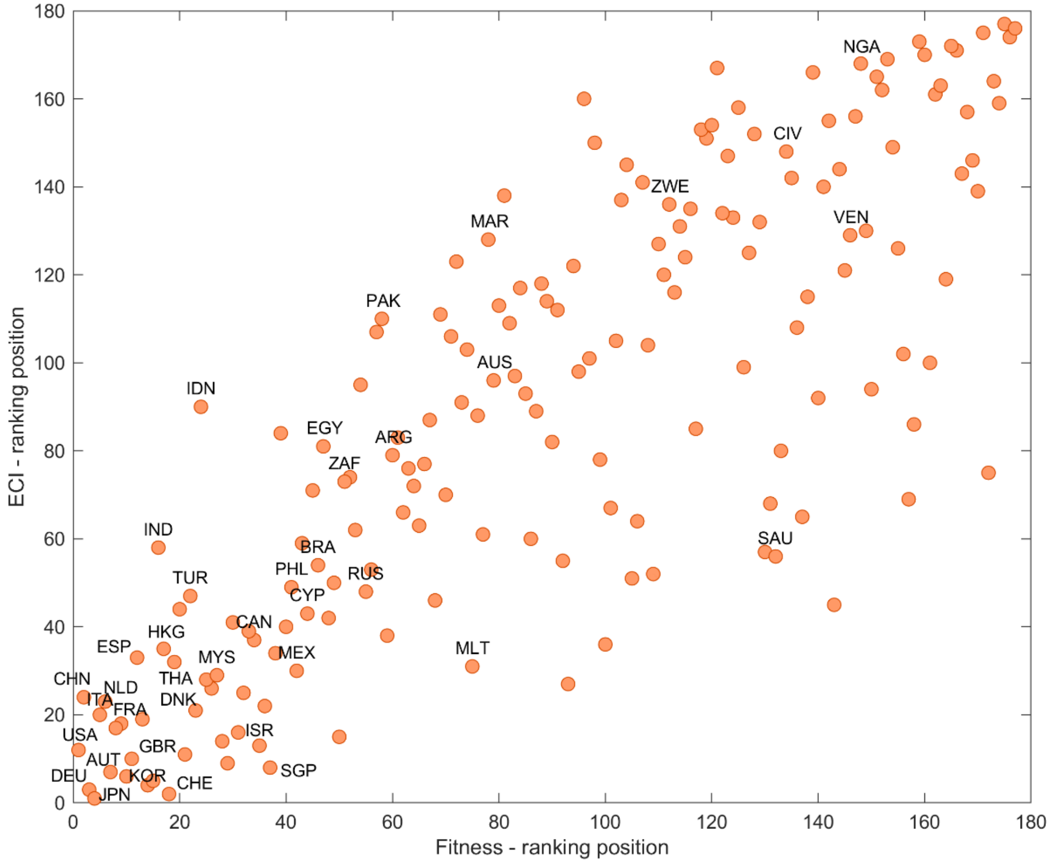


**Comparison between the rankings provided by the Fitness and the ECI values.** The rankings sort countries according to decreasing complexity, as computed by the two metrics. Results refer to year 2017.

## SUPPLEMENTARY FIGURE 2

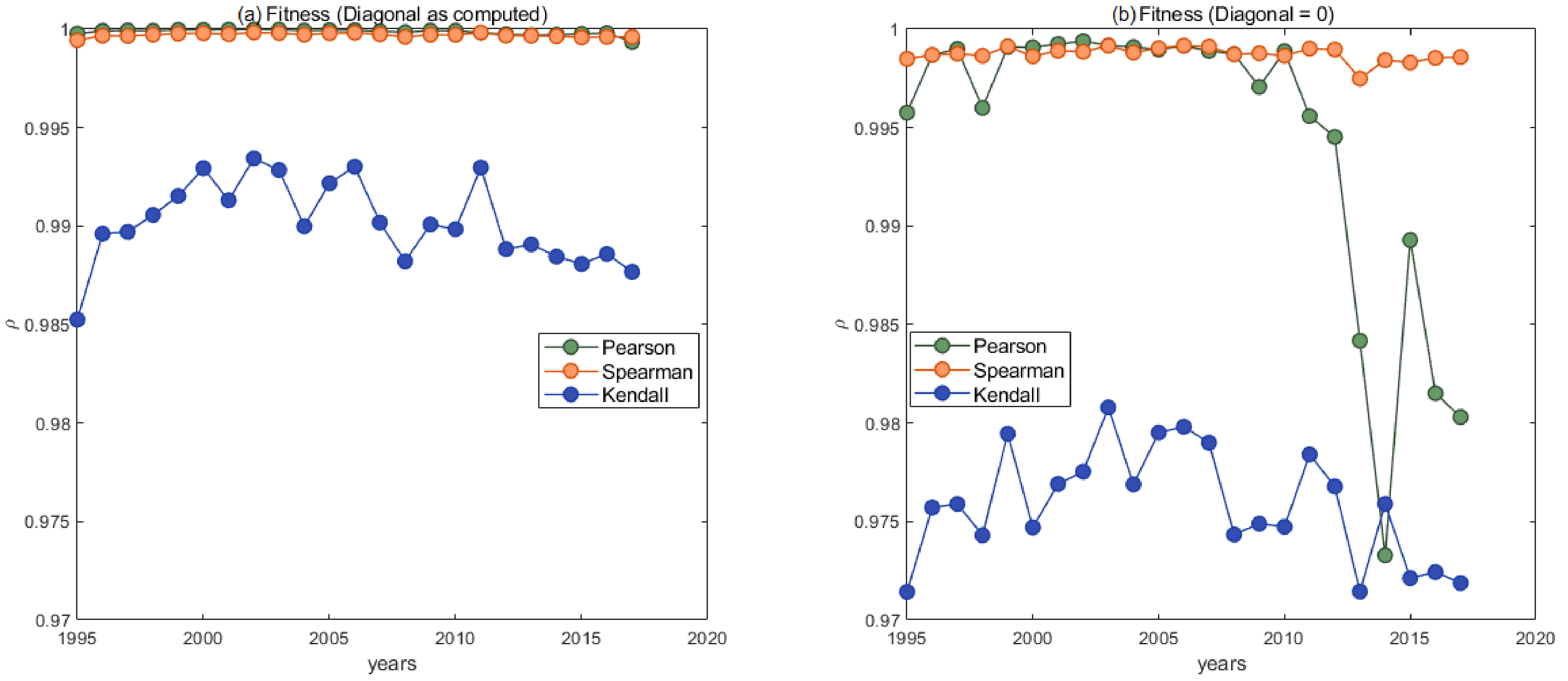


**Correlation coefficients among the non-linearly and the linearly computed values of Fitness.** In green is Pearson's correlation coefficient, while Spearman's one is in orange and Kendall's in blue. The Spearman's and Kendall's coefficients are ranking-based, while the Pearson's one compares the values between the two vectors. In panel (a), the results refer to the case in which the diagonal values of the matrix **N** are left as computed. In panel (b), the results refer to the case in which the diagonal values of the matrix **N** are set to zero. The FC iterative method has been implemented according to the directives in *(1)* (see Methods, Eqs 11). The linearised algorithm almost perfectly reproduces the outcomes of the non-linear one, whether or not the diagonal values are modified from the computed ones (panels (a) and (b), respectively; see Methods, Eq 15).

## SUPPLEMENTARY FIGURE 3

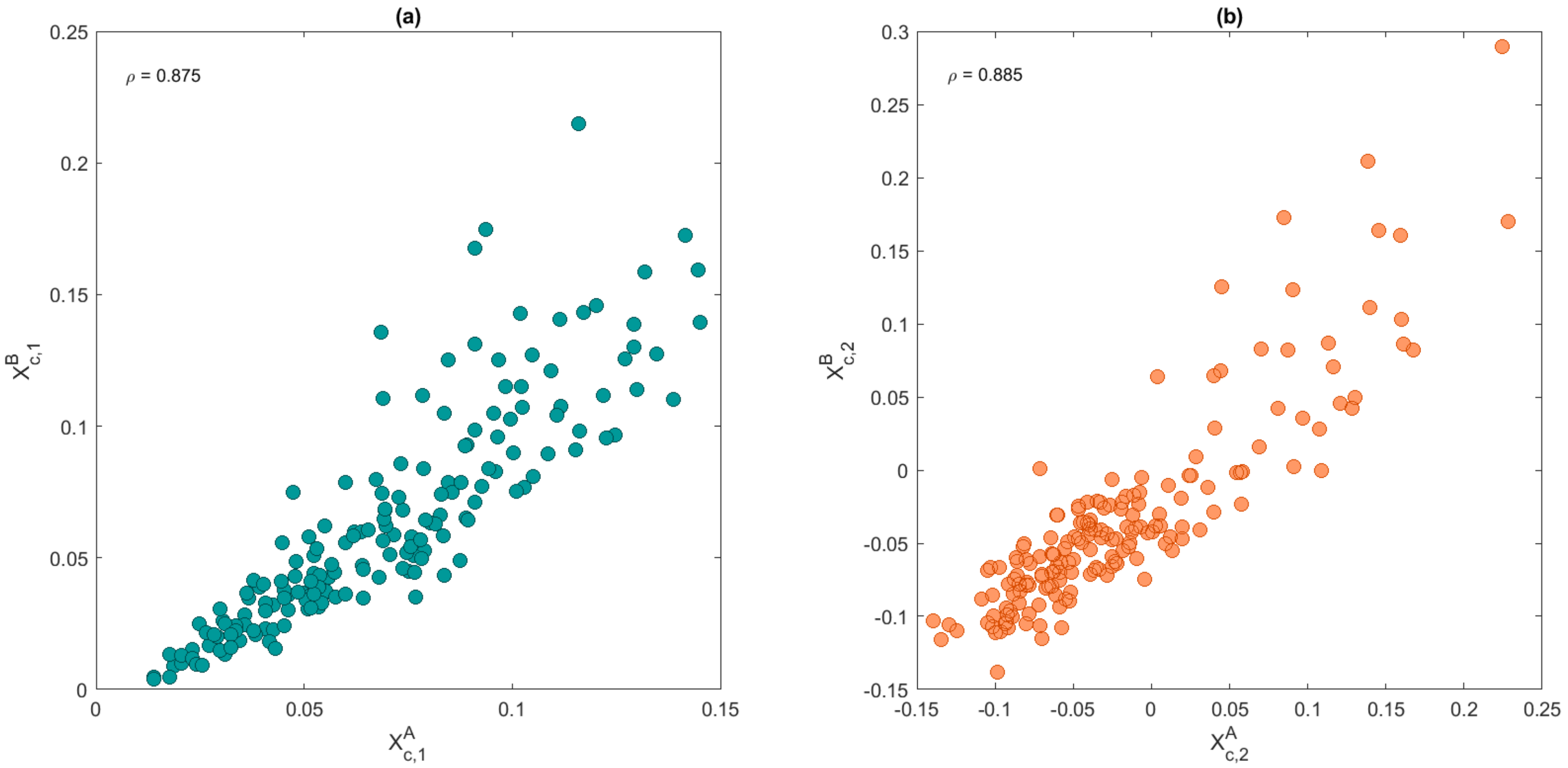


**Scatter plots comparing the eigenvectors of the proximity matrices $N^A$ and $N^B$** . The matrices are computed using the transformation matrices $W^A_{cp} = \frac{M_{cp}}{\sqrt{k_c k_p}}$ and $W^B_{cp} = \frac{M_{cp}}{k_c k'_p}$ respectively. The left plot (a) compares the eigenvectors $X^A_{c,1}$ and $X^B_{c,1}$ associated to the largest eigenvalues $\lambda^A_1$ and $\lambda^B_1$ of the two proximity matrices, $N^A$ and $N^B$. The right plot (b) compares the eigenvectors corresponding to the second largest eigenvalues $\lambda^A_2$ and $\lambda^B_2$, namely $X^A_{c,2}$ and $X^B_{c,2}$. The eigenvectors are normalized such that the Frobenius norm is unitary, i.e., $\sqrt{\sum_c X^2_{c,i}} = 1,\ (i = 1{,}2)$. As detailed in the main text, and as the correlation coefficients highlight, the eigenvectors from the two matrices carry similar information. The correlation coefficients are of the Pearson's kind.

## SUPPLEMENTARY FIGURE 4

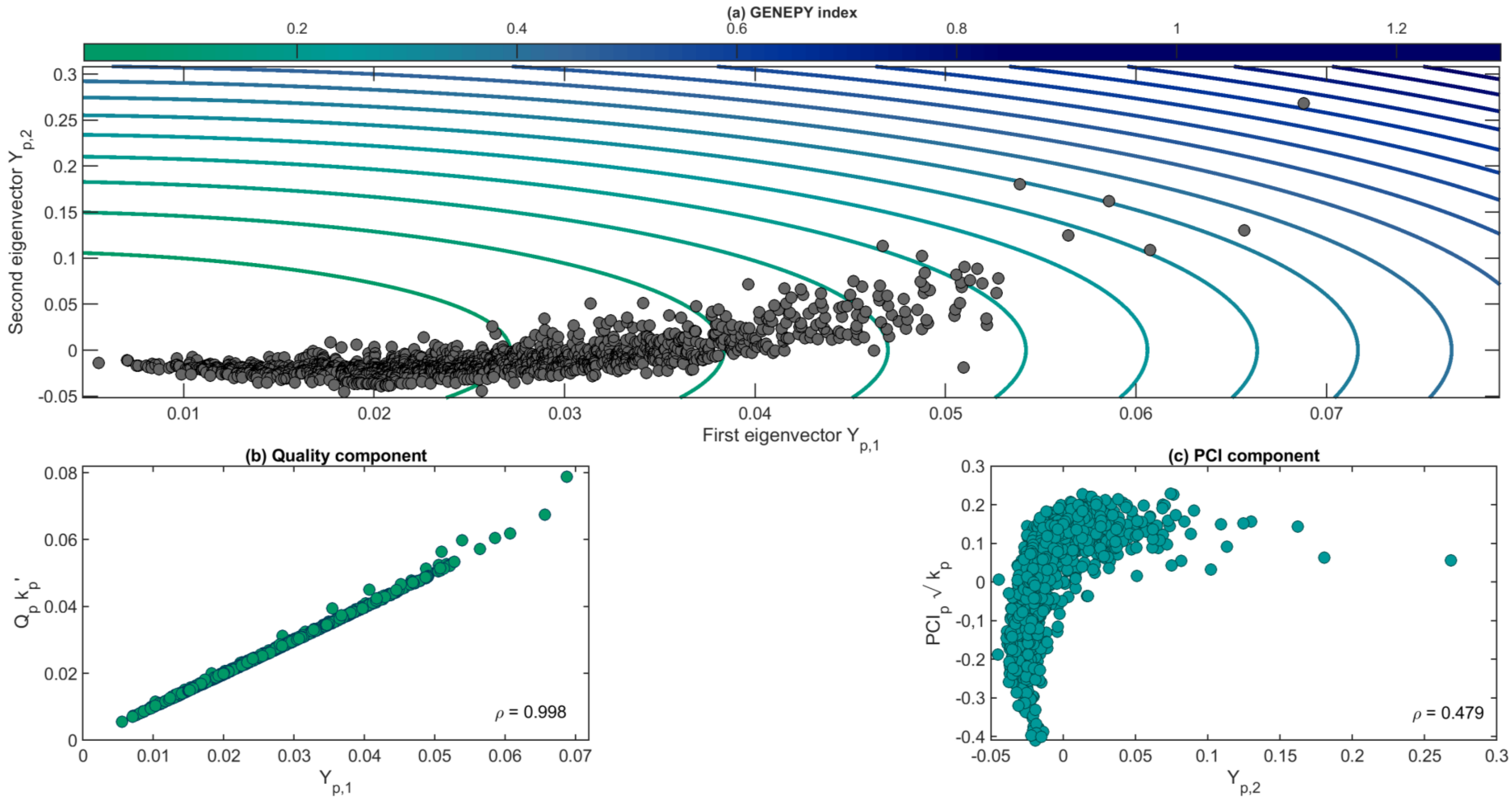


**Products' GENEPY results and components as referred to the 2017 international commodities trade.** (a) Contour plot of the GENEPY values. We interpret increasing economic complexity with increasing radial distance from the origin. The x-axis reports the values of the first eigenvector $Y_{p,1}$ whilst the y-axis the values of the second eigenvector $Y_{p,2}$. The eigenvectors are normalized such that their Frobenius norm is unitary, i.e., $\sqrt{\sum_p Y_{p,i}^2} = 1$, with $(i = 1{,}2)$. Contours range from lower GENEPY values (green) to higher ones (blue). (b) Scatter plot of the first component $Y_{p,1}$ of GENEPY values compared with the values of the Quality values $Q_p$ rescaled by the products corrected degree $k_p'$. (c) Scatter plot of the second component $Y_{p,2}$ of GENEPY compared with PCI values, rescaled by the term $\sqrt{k_p}$. The correlation coefficients are of the Pearson's kind. This year totaled 1232 traded commodities.

## SUPPLEMENTARY FIGURE 54

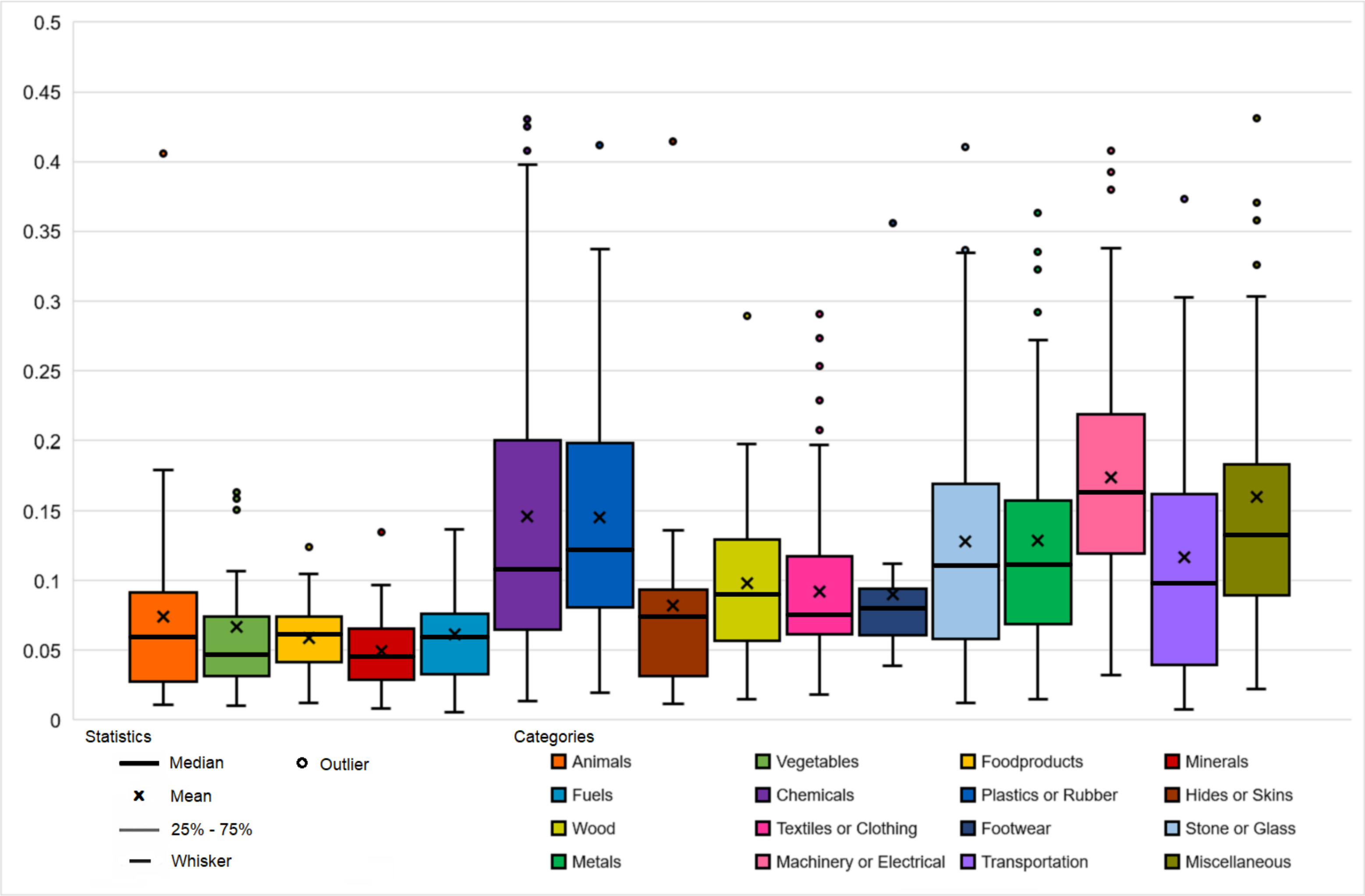


**Boxplots of the GENEPY values for products aggregated into categories.** Categories are defined as according to *(6)*. In the boxplot the cross is the mean, the thick bar is the median, the bars define the interquartile range (IQR) 25% - 75%, the shorter bars are the whiskers and the dots are outliers. From above the upper quartile, a distance of 1.5 times the IQR is measured out and a whisker is drawn up to the largest observed point from the dataset that falls within this distance. Similarly, a distance of 1.5 times the IQR is measured out below the lower quartile and a whisker is drawn up to the lower observed point from the dataset that falls within this distance. All other observed points are plotted as outliers.

## SUPPLEMENTARY FIGURE 6

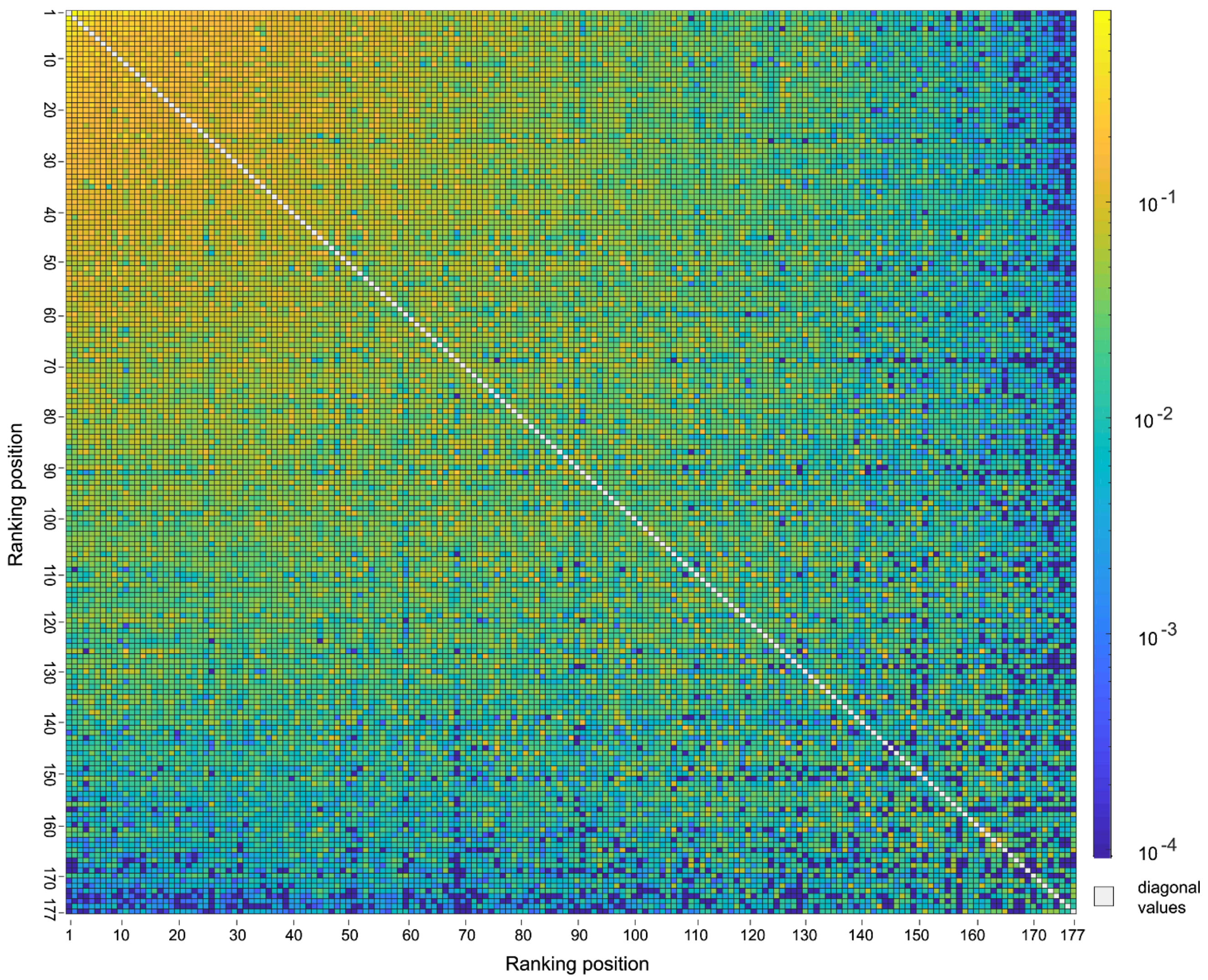


**The elements $N_{cc^*}$ of the similarity matrix N for the 2017 trade**. Rows and columns reordered top-to-bottom, left-to-right, according to decreasing values of GENEPY complexity. More complex countries are found on the top (left) of the matrix. Correspondence among ranking positions and countries are defined in Supplementary Table 1.

## Supplementary Figure 7

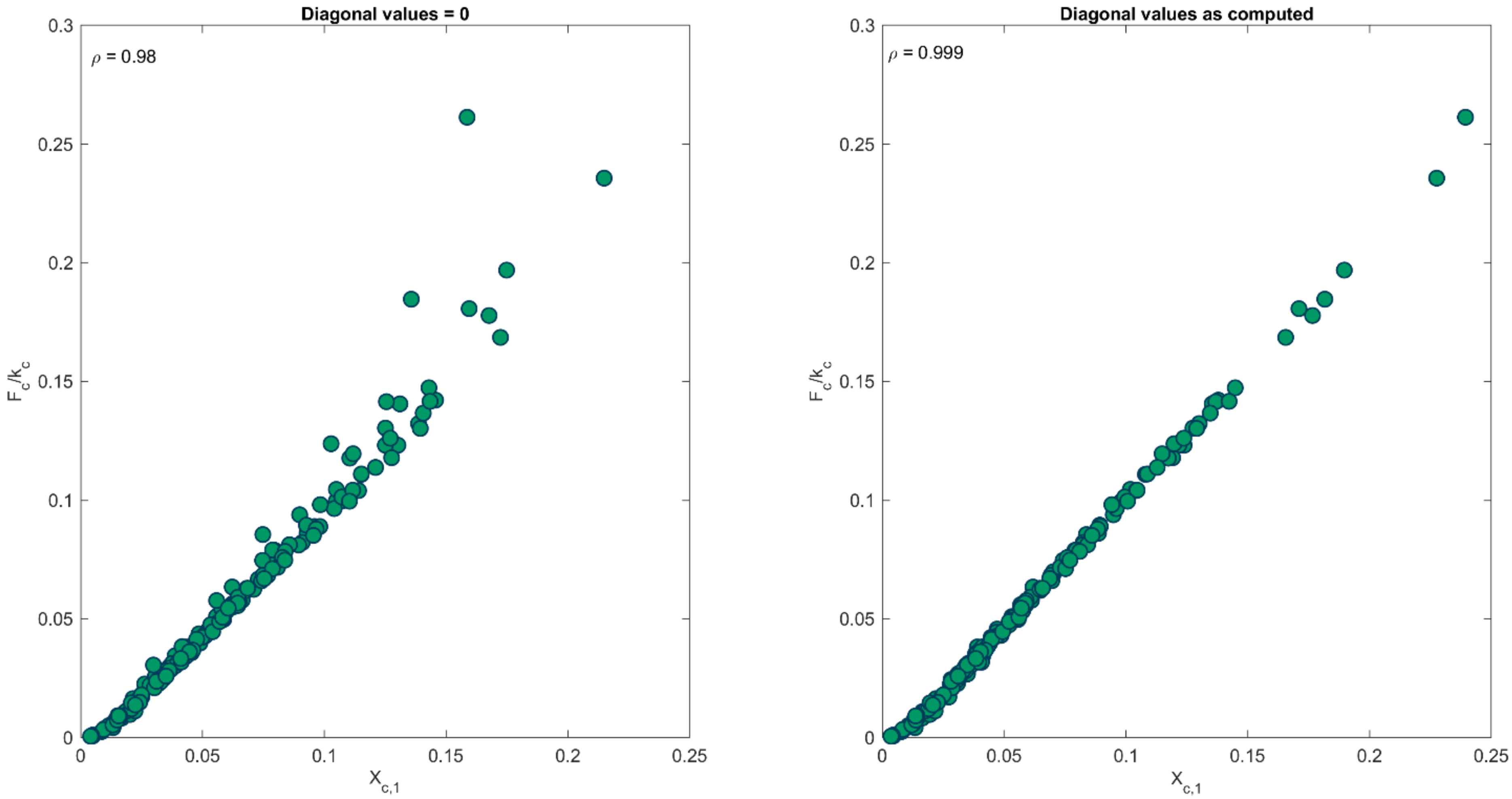


**Scatter plots of the eigenvectors $X_{c,1}$ and $F_c/k_c$ for different interpretations of the matrix N**. On the left, the eigenvector $X_{c,1}$ belongs to the matrix $N$ with diagonal values set to zero. On the right, $X_{c,1}$ is the eigenvector of the matrix $N$ in which we left the diagonal values as computed, i.e., $N_{cc} = \sum_p M_{cp} M_{c^*p} \,/\, k_c k_c (k'_p)^2$ (see Methods, Eq 15). Data refer to year 2017.

## Supplementary Figure 8

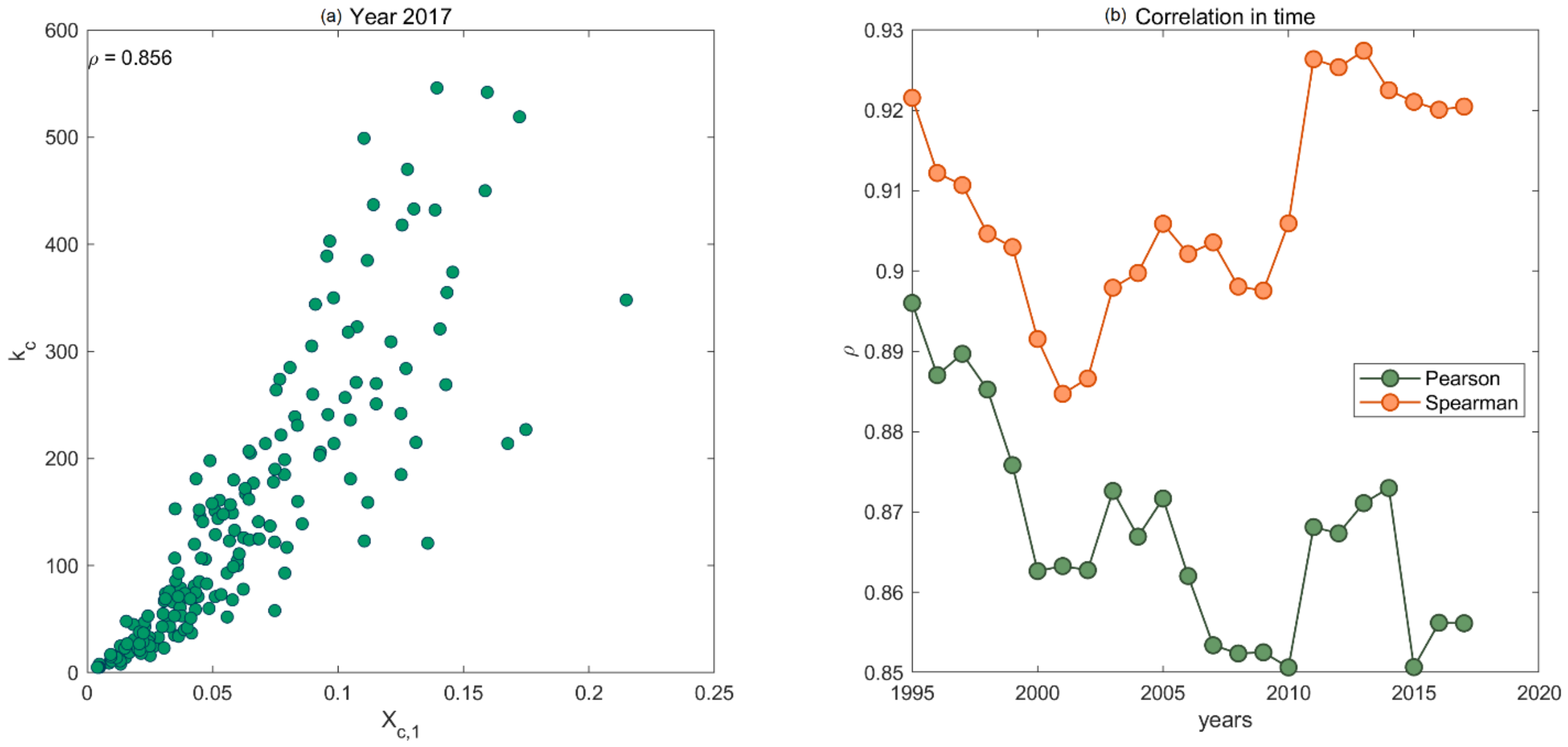


**Correlation between $X_{c,1}$ and $k_c$.** In panel (a), the scatter plot of the values from year 2017. In panel (b), the values of the correlation coefficients between the two vectors during time. The correlation of the Pearson's kind is in green, while the Spearman's one in orange.

## SUPPLEMENTARY FIGURE 9

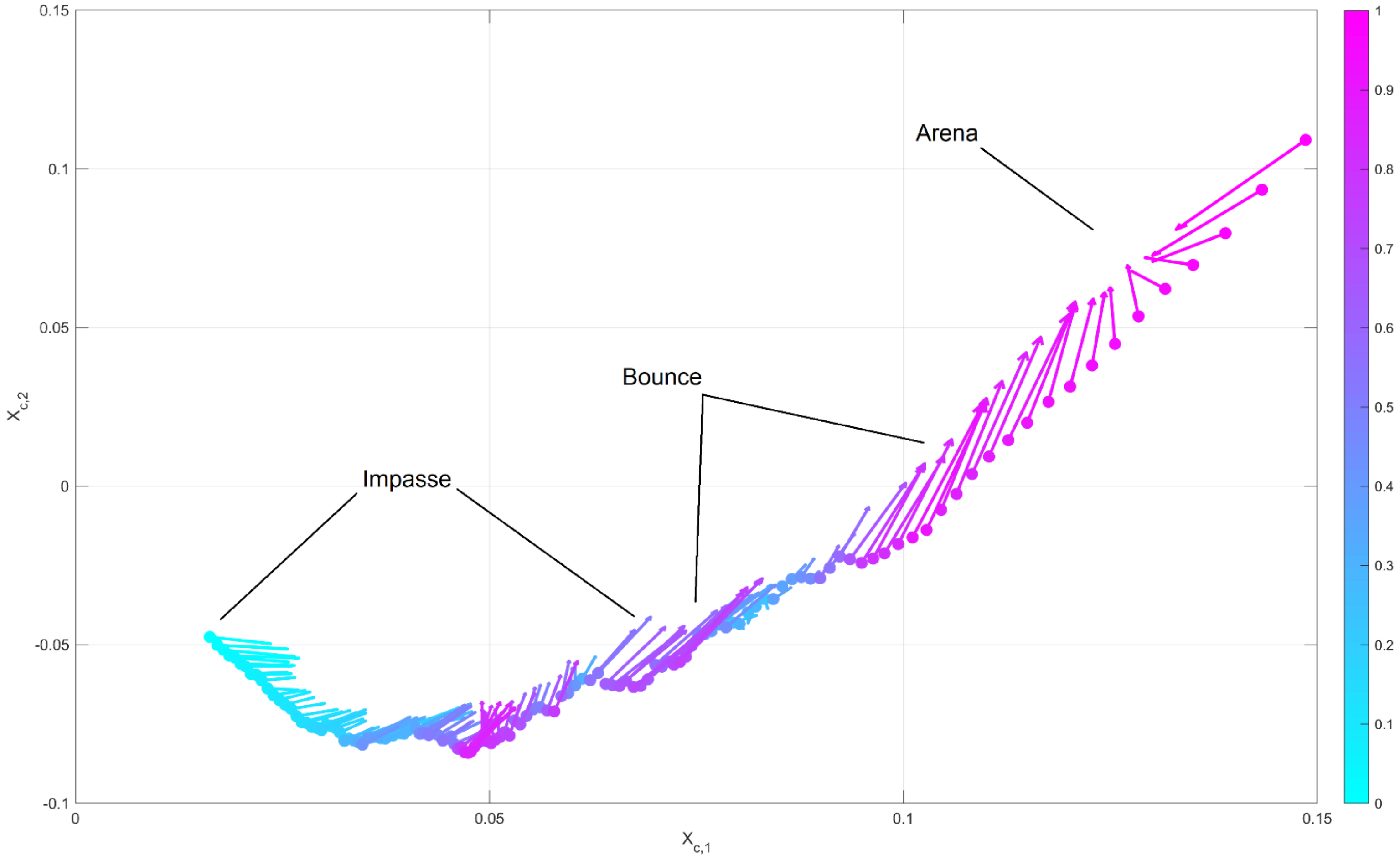


**The time regimes of economic growth according to the two contributions $X_{c,1}$ and $X_{c,2}$.** During time, countries move along the knee-like shape designed by the arrows.

## SUPPLEMENTARY FIGURE 10

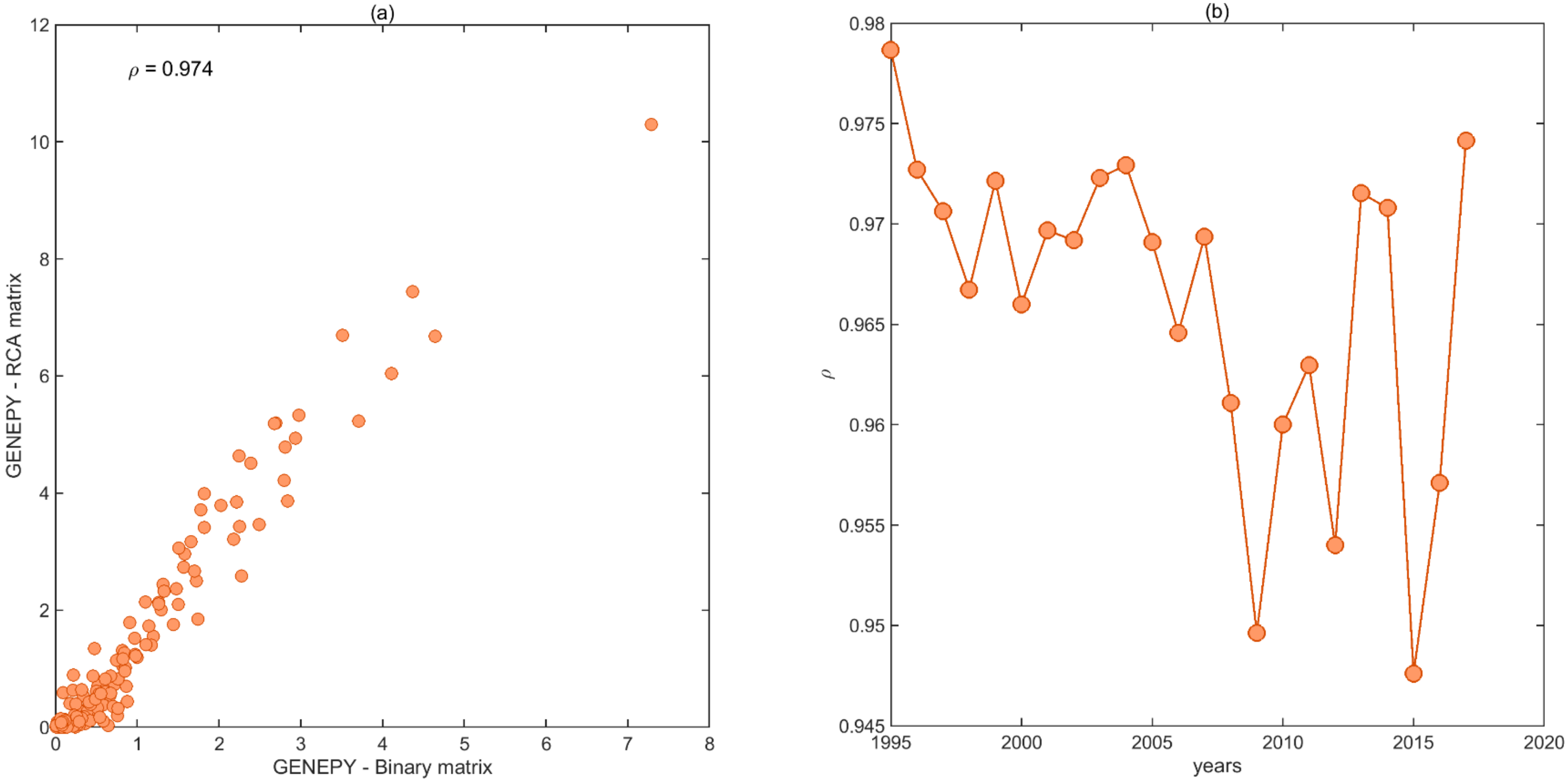


**Comparison of the GENEPY of countries computed using either binary or RCA matrix.** (a) Scatter plot of the GENEPY index as obtained from the use of the binary matrix M – on the x-axis – and from the RCA matrix – on the y-axis – as input for the computation of the GENEPY values. Values refer to year 2017. In panel (b), time series of the correlation coefficients among the GENEPY values computed using as input for the algorithm the binary matrix M and the ones obtained using as input the RCA matrix. The correlation coefficients are of the Pearson's kind.

## SUPPLEMENTARY FIGURE 11

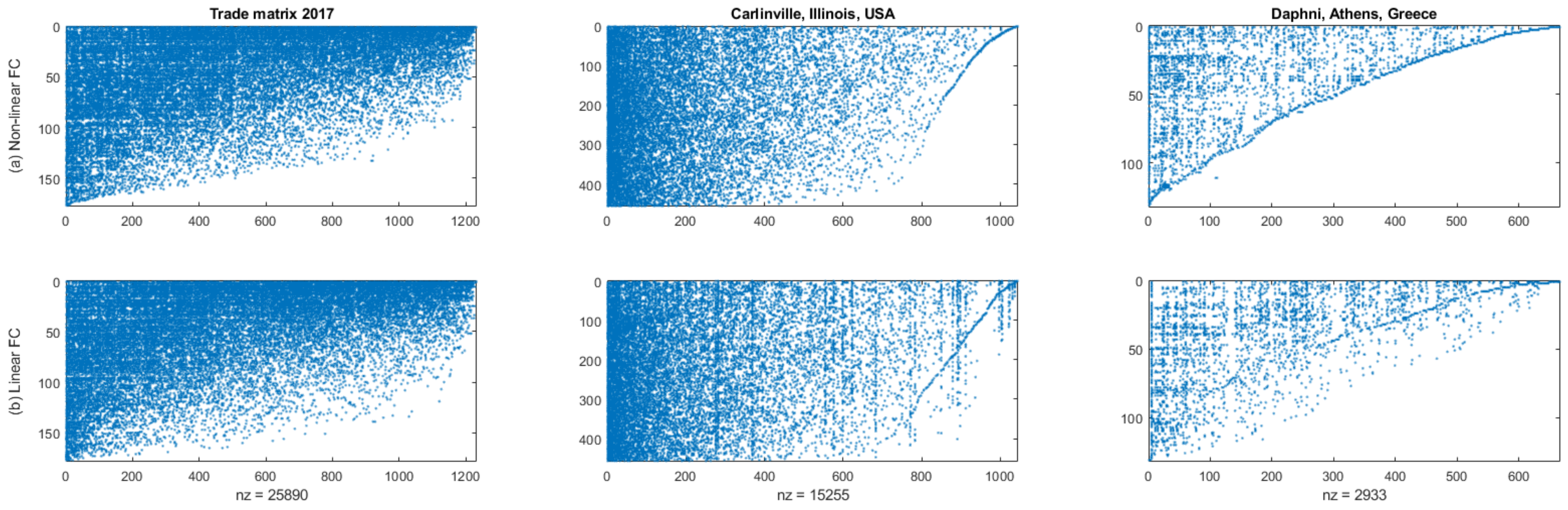


**Nestedness maximization performances.** Visual comparison among the performances of the non-linear FC algorithm (panels (a)) and its linearized form, (panels (b)) in maximizing nestedness of matrices. The left panels refer to the countries-products bipartite network during 2017. The central panels refer to the network of pollination in Carlinville, Illinois, USA (network ID: M_PL_062); on the right the one referring to the pollination in Daphní, Athens, Greece (network ID: M_PL_015). Data for the pollination networks are freely available at www.web-of-life.es.

# SUPPLEMENTARY TABLES

## SUPPLEMENTARY TABLE 1

**Ranking positions of countries according to ascending values of GENEPY**. Results refer to year 2017.

| Ranking position | Country iso-3 code | Country name | Ranking position | Country iso-3 code | Country name | Ranking position | Country iso-3 code | Country name |
|---|---|---|---|---|---|---|---|---|
| 1 | JPN | Japan | 60 | VGB | British Virgin Islands | 119 | CUQ | Curaçao |
| 2 | KOR | Korea, Rep. of Korea | 61 | COL | Colombia | 120 | SLE | Sierra Leone |
| 3 | DEU | Germany | 62 | LBN | Lebanon | 121 | RWA | Rwanda |
| 4 | CHE | Switzerland-Liechtenstein | 63 | PRK | Korea, Dem. People's Rep. of | 122 | TGO | Togo |
| 5 | USA | United States of America | 64 | PAK | Pakistan | 123 | NIC | Nicaragua |
| 6 | CHN | China | 65 | URY | Uruguay | 124 | ETH | Ethiopia |
| 7 | CZE | Czech Republic | 66 | LKA | Sri Lanka | 125 | MOZ | Mozambique |
| 8 | GBR | United Kingdom | 67 | ARG | Argentina | 126 | SAU | Saudi Arabia |
| 9 | HKG | Hong Kong (SARC) | 68 | BRB | Barbados | 127 | TJK | Tajikistan |
| 10 | SWE | Sweden | 69 | KHM | Cambodia | 128 | BDI | Burundi |
| 11 | SGP | Singapore | 70 | MKD | The former Yugoslav Rep. of Macedonia | 129 | BTN | Bhutan |
| 12 | AUT | Austria | 71 | MDA | Moldova, Rep.of | 130 | KWT | Kuwait |
| 13 | ITA | Italy | 72 | KGZ | Kyrgyzstan | 131 | BEN | Benin |
| 14 | MYS | Malaysia | 73 | JOR | Jordan | 132 | MWI | Malawi |
| 15 | BEL | Belgium-Luxembourg | 74 | UZB | Uzbekistan | 133 | GHA | Ghana |
| 16 | ISR | Israel | 75 | SYR | Syrian Arab Republic | 134 | CMR | Cameroon |
| 17 | THA | Thailand | 76 | KAZ | Kazakstan | 135 | AZE | Azerbaijan |
| 18 | FRA | France | 77 | MUS | Mauritius | 136 | CUB | Cuba |

| Ranking position | Country iso-3 code | Country name | Ranking position | Country iso-3 code | Country name | Ranking position | Country iso-3 code | Country name |
|---|---|---|---|---|---|---|---|---|
| 19 | NLD | Netherlands | 78 | CRI | Costa Rica | 137 | CIV | Côte d'Ivoire |
| 20 | FIN | Finland | 79 | AUS | Australia | 138 | TTO | Trinidad and Tobago |
| 21 | SVN | Slovenia | 80 | MNE | Montenegro | 139 | GIB | Gibraltar |
| 22 | SVK | Slovakia | 81 | CHL | Chile | 140 | BHS | Bahamas |
| 23 | HUN | Hungary | 82 | DOM | Dominican Republic | 141 | COD | Democratic Republic of the Congo |
| 24 | POL | Poland | 83 | PER | Peru | 142 | SDN | Sudan (2011) |
| 25 | PHL | Philippines | 84 | ARE | United Arab Emirates | 143 | DZA | Algeria |
| 26 | IRL | Ireland | 85 | ARM | Armenia | 144 | TKM | Turkmenistan |
| 27 | IND | India | 86 | ALB | Albania | 145 | MHL | Marshall Islands |
| 28 | ESP | Spain | 87 | ABW | Aruba | 146 | NER | Niger |
| 29 | DNK | Denmark | 88 | MMR | Myanmar | 147 | GMB | Gambia |
| 30 | ROU | Roumania | 89 | MAR | Morocco | 148 | GUY | Guyana |
| 31 | MEX | Mexico | 90 | SYC | Seychelles | 149 | NCL | New Caledonia |
| 32 | HRV | Croatia | 91 | BGD | Bangladesh | 150 | PNG | Papua New Guinea |
| 33 | EST | Estonia | 92 | FJI | Fiji | 151 | BRN | Brunei Darussalam |
| 34 | IDN | Indonesia | 93 | PRY | Paraguay | 152 | VEN | Venezuela |
| 35 | VNM | Viet Nam | 94 | ISL | Iceland | 153 | SUR | Suriname |
| 36 | LTU | Lithuania | 95 | KEN | Kenya | 154 | NGA | Nigeria |
| 37 | PRT | Portugal | 96 | GEO | Georgia | 155 | BFA | Burkina Faso |
| 38 | TUR | Turkey | 97 | BHR | Bahrain | 156 | SOM | Somalia |
| 39 | BLR | Belarus | 98 | ATG | Antigua and Barbuda | 157 | ERI | Eritrea |
| 40 | BRA | Brazil | 99 | MDG | Madagascar | 158 | COG | Congo |
| 41 | CYP | Cyprus | 100 | JAM | Jamaica | 159 | YEM | Yemen |
| 42 | BGR | Bulgaria | 101 | LAO | Lao People's Democratic Republic | 160 | GIN | Guinea |
| 43 | LVA | Latvia | 102 | GTM | Guatemala | 161 | QAT | Qatar |
| 44 | CAN | Canada | 103 | HND | Honduras | 162 | LBR | Liberia |
| 45 | NOR | Norway | 104 | UGA | Uganda | 163 | VUT | Vanuatu |
| 46 | UKR | Ukraine | 105 | OMN | Oman | 164 | SLB | Solomon Islands |

| Ranking position | Country iso-3 code | Country name | Ranking position | Country iso-3 code | Country name | Ranking position | Country iso-3 code | Country name |
|---|---|---|---|---|---|---|---|---|
| 47 | TUN | Tunisia | 106 | SEN | Senegal | 165 | GAB | Gabon |
| 48 | RUS | Russian Federation | 107 | IRN | Iran (Islamic Republic of) | 166 | MRT | Mauritania |
| 49 | SRB | Serbia | 108 | BLZ | Belize | 167 | FLK | Falkland Islands (Malvinas) |
| 50 | NPL | Nepal | 109 | ZMB | Zambia | 168 | GRL | Greenland |
| 51 | MAC | Macau | 110 | MNG | Mongolia | 169 | TCD | Chad |
| 52 | SLV | El Salvador | 111 | HTI | Haiti | 170 | CYM | Cayman Islands |
| 53 | ZAF | South Africa | 112 | BOL | Bolivia | 171 | MDV | Maldives |
| 54 | MLT | Malta | 113 | VCT | Saint Vincent and the Grenadines | 172 | LBY | Libyan Arab Jamahiriya |
| 55 | BIH | Bosnia and Herzegovina | 114 | ZWE | Zimbabwe | 173 | AGO | Angola |
| 56 | EGY | Egypt | 115 | AFG | Afghanistan | 174 | GNB | Guinea-Bissau |
| 57 | GRC | Greece | 116 | ECU | Ecuador | 175 | SSD | South Sudan |
| 58 | NZL | New Zealand | 117 | TZA | Tanzania, United Rep. of | 176 | IRQ | Iraq |
| 59 | PAN | Panama | 118 | MLI | Mali | 177 | GNQ | Equatorial Guinea |

# SUPPLEMENTARY NOTES

## SUPPLEMENTARY NOTE 1: IMPLEMENTATION OF THE ALGORITHMS

The two algorithms of economic complexity, i.e., the *Method of Reflection*, MR, and the *Fitness and Complexity* algorithm, FC, have been separately implemented following the steps presented in Sect. *Methods*. In order to prevent divergence to null or infinite values, our implementation of the FC algorithm in Eqs 11 of the main text includes the convergence criteria adopted in (1): the algorithm stops at the iteration *N*, when the rankings between step *N* and step *N+ΔN*, have a Spearman's correlation coefficient larger than *0.999*, in this way ensuring *rank convergence* of the algorithm. In this work we assumed *ΔN=10.*

Small divergences of the results with respect to the official ones provided by the World Bank (2) – for the Fitness values – and from the Observatory of Economic Complexity (3) – for ECI – should be attributed to either different sanitation procedure of the data, also considering the larger number of countries here accounted for, *or* differences in the convergence criteria adopted for the FC algorithm.

## SUPPLEMENTARY NOTE 2: THE COMPLEXITY OF PRODUCTS

Supplementary Figures 4 – 5 show the results of the GENEPY index for products. In Supplementary Fig. 4, panel (b), higher correlation is found among $Y_{p,1}$ and the term $Q_p k'_p$ , in fact we showed how the first eigenvector $Y_{p,1}$ solves the problem of finding linearly computed Quality values, as expected. Lower correlation is found when comparing the values of the second eigenvectors $Y_{p,2}$ with the values $PCI_p\,\sqrt{k_p}$ , panel (c), due to the differences among the matrix $W^A_{cp} = M_{cp}/\sqrt{k_c}\sqrt{k_p}$, from which we are able to recover the exact metrics ECI and PCI, and the matrix $W^B_{cp} = M_{cp}/k_c\, k'_p$ , which are here used to compute the GENEPY (hence $Y_{p,1}$ and $Y_{p,2}$) values. In Supplementary Fig. 5, differences in the complexity computed according to the GENEPY index among the categories clearly emerge: commodities into the *Machinery* or *Electrical* categories naturally require different and more sophisticated knowledge in order to be produced, while resource-based commodities, such as *Animals* or *Foodproducts* do not need special knowledge requirements in order to be produced or traded. In addition, the GENEPY values may vary widely in some categories such as *Chemicals*, where the natural availability of natural resources and the requirements for their extraction may define the need for higher complex technologies for making these available for trade.

## SUPPLEMENTARY NOTE 3: THE KNEE-LIKE SHAPE

The knee-shape of the points in the plane $X_{c,1} - X_{c,2}$ is recurrent in all the years of analysis, thus showing the existence of a functional relationship between the two eigenvectors. The reasons of the knee-like shape of this functional relationship are related to linear algebra and network science.

Let us define a functional relationship $\mathrm{f}$ between $X_{c,1}$ and $X_{c,2}$ s.t.

$$X_{c,2} = f\left(X_{c,1}\right) + \epsilon_c, \qquad \text{(Eq 1)}$$

where $\epsilon_c$ are the errors. We assume the errors to have null expected value, i.e., $\mathrm{E}(\epsilon_c) = 0$ and to be orthogonal to $X_{c,1}$, s.t., $\sum_c X_{c,1}\epsilon_c = 0.$

There exist some constraints related to the existence of the eigenvectors of a symmetric matrix, which any functional relationship should respect:

(i) the eigenvectors corresponding to distinct eigenvalues of a symmetric squared matrix are, by definition, orthogonal and this entails that the inner product of the vectors is zero, i.e., $\sum_c X_{c,1} \cdot X_{c,2} = 0$;

(ii) for the Perron-Frobenius theorem, the eigenvector corresponding to the largest eigenvalue is strictly positive, s.t. $X_{c,1} \geq 0 \ \forall \ c = 1, \ldots, C$ (number of countries);

(iii) we can normalize the eigenvectors such that the 2-norm is unitary, i.e., $\sum_c X_{c,1}^2 = \sum_c X_{c,2}^2 = 1$;

(iv) if any element of the eigenvector corresponding to the first (largest) eigenvalue $\lambda_1$ is zero, the same element is null also within the successive eigenvectors. In fact, the eigen-equation for the matrix $\mathrm{N}$ is:

$$X_{c^*,1}\lambda_1 = \sum_c N_{cc^*} X_{c,1};$$

because of condition (ii), it holds that $X_{c^*,1} = 0$ iff $\sum_c N_{cc^*} = 0$, i.e., if the matrix has null elements all along the column (or row) $c^*$. Interpreting this result through network science lenses, the node to which the null element of the eigenvector refers is disconnected in the network. Therefore, in the hypothesis of existence of any functional relationship between two eigenvectors as in Eq 1, it must hold $f(0) = 0$.

We now proceed exploring two cases of possible functional relationship for Eq 1.

**CASE A**: The simplest form of this relation considers f as a linear function, i.e.,

$$X_{c,2} = aX_{c,1} + \epsilon_c \qquad \text{(Eq 2)}$$

By imposing the orthogonality condition (i) to Eq 2 one obtains:

$$\sum_c X_{c,1}X_{c,2} = \sum_c X_{c,1}(aX_{c,1} + \epsilon_c) = a\sum_c X_{c,1}^2 + \sum_c X_{c,1}\epsilon_c = 0$$

Since the errors are orthogonal to $X_{c,1}$ and the 2-norm of the vector is unitary for condition (iii), the solution is $a = 0$, which entails no functional relationship exists between $X_{c,1}$ and $X_{c,2}$.

**CASE B**: We consider the function to be polynomial of the second order, namely:

$$X_{c,2} = aX_{c,1} + bX_{c,1}^2 + \epsilon_c \qquad \text{(Eq 3)}$$

Again, by applying the orthogonality condition (i), one has:

$$\sum_c X_{c,1}X_{c,2} = \sum_c X_{c,1}(aX_{c,1} + bX_{c,1}^2 + \epsilon_c) = a\sum_c X_{c,1}^2 + b\sum_c X_{c,1}^3 + \sum_c X_{c,1}\epsilon_c = 0$$

which leads to

$$a + b\sum_c X_{c,1}^3 = 0 \qquad \text{(Eq 4)}$$

Because of condition (ii), the term $\sum_c X_{c,1}{}^3$ is strictly positive and in order to respect Eq 4, the values of the parameter $a$ and $b$ should have different signs, thus justifying the existence of the knee-like shape. In particular, the upward belly of the relation is given for negative values of the parameter $a$ and positive values of $b$. In this sense, the minimum point depends on the parameters.

We can read this result through the meaning of the matrix and its eigenvectors in the context of network theory. In fact, in this case the eigenvectors of the matrix describe the structural properties of the network (4) and are related to the similarity of the network among the countries. In fact, the shape of the matrix $\mathrm{N}$ (Figure S4 of this SI), represents a connected network in which a stronger connected component can be spotted, constituted by the top-GENEPY countries, while weaker connections characterize the countries at the periphery. In this weak connection component, as shown in (5), the correlation between the two eigenvectors is positive. Also, as stated by the authors in (5), the mutual signs of the elements of the eigenvectors corresponding to the two largest eigenvalues – whether these are positive or negative in the second eigenvector – acquires a meaning, thus justifying the presence of three areas (or groups) in which the points can stand:

i) both values $X_{c,1}$ and $X_{c,2}$ are low: these nodes belong to the weaker component and they have no important connections with the strongest connected component;

ii) both values $X_{c,1}$ and $X_{c,2}$ are high: these nodes belong to the strongest and more connected core of the network, thus defining the area in which these points (countries) are competitors, also in the sense of collecting most of the links in terms of similarities;

iii) low values of $X_{c,1}$ , high values of $X_{c,2}$ or viceversa: this situation identifies the presence of some “outliers” of the core and the periphery components. These nodes connect the stronger and the weaker components and have a role in bridging the gaps across the network. We identify these nodes as able to jump, during time, from one group to another.

These three behaviors along the knee-like shape evolve in time, letting the dynamical regimes of growth *Impasse, Bounce* and *Arena,* emerge as we analyse the aggregated dynamics of countries in time.

## SUPPLEMENTARY NOTE 4: THE REGIMES OF GROWTH

An analysis of the trajectories of countries according to their values in $X_{c,1}$ and $X_{c,2}$ allows one distinguishing the presence of the three regimes of growth along the knee-like shape: *Impasse, Bounce* and *Arena.*

For each country whose continuous data in time are available (154 countries), we defined the main displacement recorded by the country identifying a starting and an ending point during the period of analysis. We connect the point located at the center of mass of $\mathrm{X}_{c,1}$ and $\mathrm{X}_{c,2}$ during the first 3 years of analysis (1995 – 1998) to the center of mass during the last 3 years (2014 – 2017). In order to make the overall dynamics clearer, we defined overlapping classes of countries using a moving window of 20 countries per each class. Firstly, we ordered the countries (and respective scores of the eigenvectors in time) for increasing starting $\mathrm{X}_{c,1}$ values.

Secondly, by defining each class through a window of 20 countries, we computed the resultant vector of the displacements of the countries falling in that class. Lastly, we applied the resultant vectors to the barycenter of the starting points of the single vectors that fall into the class.

In Supplementary Figure 9, we show the aggregated dynamics of countries along the knee-shape. The colours sort the vectors for their length, as normalized for the longest vector recorded (light blue identifies the shorter ones, light purple the longer ones). The light blue vectors on the bottom left part of the knee identify the *Impasse*: the dynamics of the countries in this area are here tangled, as shown by the horizontal displacement of the vectors. Notwithstanding the presence of some uplift movements of the classes around the minimum point in $X_{c,1} = 0.05$, the countries within this area are stacked in this dynamic of poor diversification and complexity within the cluster of lower growth. As soon as countries reinforce their knowledge, the countries experience higher values of $X_{\mathrm{c},2}$ until these values approach to zero: here it starts the *Bounce*, where countries boost their diversification and complexity, turning cluster membership by joining the more economically grown countries club and thus increasing the similarity in the export basket with them. Longer vectors in violet and light purple highlight the jump. Once the economies have experienced the boost, they join the *Arena* of competition, for which continuous growth is determined. It is interesting to observe a divergent direction in the highest part of the Arena (high values of $X_{c,1}$ and $X_{c,2}$). Here, countries may lose ground on the plane of growth. Many factors may contribute to this downgrading dynamic. In fact, as described in the main article, the economic and financial crisis are more likely to be the cause of these drops; also, the entrance in the markets of new economies decreases the potential of economies to increase their economic complexity in time.

## SUPPLEMENTARY NOTE 5: ECOLOGICAL NETWORKS

As detailed in the Discussion, the very similar results between the linear and the non-linear versions of the FC algorithm cannot be generalised to other systems. In fact, non-linearity has been shown to be an important feature of the algorithms for temperature minimization and the FC algorithm has very good potential in minimizing the nestedness temperature of ecological networks (1). Therefore, we have tested the packing performance of the linearized form of the FC algorithm, similarly to the comparison showed in (1). We exemplify the results through the analysis of two pollination networks provided by The Web of Life project and available at www.web-of-life.es (network IDs : M_PL_062 and : M_PL_015). The networks describe the pollination phenomena among plants and pollinators. As Supplementary Figure 11 shows, the non-linear algorithm outperforms the linearised form in its capability of maximizing the nestedness of the incidence matrices of the two pollination. Instead, there are no significant differences between the non-linear and the linear algorithm for maximising the data-packing of the trade matrix, confirming that the feature of linearity pertains to the countries-products bipartite network as far as explored.

# Supplementary References


1. **Lin, J. H., Tessone, C. and Mariani, M.** Nestedness maximization in complex networks through the fitness-complexity algorithm. 2018.

2. **Tacchella, A., et al.** Economic Fitness - The World Bank. [Online] 2018. datacatalog.worldbank.org/dataset/economic-fitness.

3. **Hidalgo, C. A. and Hausmann, R.** The Observatory of Economic Complexity. [Online] 2018. oec.world/en/.

4. **Newman, M. E.** Spectral methods for community detection and graph partitioning. 2013, Vol. 88, 4.

5. **Lucińska, M., & Wierzchoń, S. T.** Clustering based on eigenvectors of the adjacency matrix. *International Journal of Applied Mathematics and Computer Science.* 2018, 28.

6. **World Integrated Trade Solutions.** *WITS - World Bank.* [Online] wits.worldbank.org.

7. **Mealy, P., Farmer, J. D., & Teytelboym, A.** Interpreting economic complexity. 2019, Vol. 51, 1.
