# Supplementary material for: Reconciling contrasting views on economic complexity
Source: Nat Commun. 2020 Jul 3;11:3352. doi: 10.1038/s41467-020-16992-1 (PMC7335174; doi:10.1038/s41467-020-16992-1)
Supplement: Supplementary file 3 — Reporting Summary [file 41467_2020_16992_MOESM3_ESM.pdf]

nature research

Corresponding author(s): Carla Sciarra

Last updated by author(s): Jun 6, 2020

# Reporting Summary

Nature Research wishes to improve the reproducibility of the work that we publish. This form provides structure for consistency and transparency in reporting. For further information on Nature Research policies, seeAuthors & Referees and theEditorial Policy Checklist .

## Statistics

For all statistical analyses, confirm that the following items are present in the figure legend, table legend, main text, or Methods section.

| n/a | Confirmed | |
|---|---|---|
| ☒ | ☐ | The exact sample size (*n*) for each experimental group/condition, given as a discrete number and unit of measurement |
| ☒ | ☐ | A statement on whether measurements were taken from distinct samples or whether the same sample was measured repeatedly |
| ☐ | ☒ | The statistical test(s) used AND whether they are one- or two-sided<br>*Only common tests should be described solely by name; describe more complex techniques in the Methods section.* |
| ☒ | ☐ | A description of all covariates tested |
| ☒ | ☐ | A description of any assumptions or corrections, such as tests of normality and adjustment for multiple comparisons |
| ☐ | ☒ | A full description of the statistical parameters including central tendency (e.g. means) or other basic estimates (e.g. regression coefficient) AND variation (e.g. standard deviation) or associated estimates of uncertainty (e.g. confidence intervals) |
| ☒ | ☐ | For null hypothesis testing, the test statistic (e.g. *F*, *t*, *r*) with confidence intervals, effect sizes, degrees of freedom and *P* value noted<br>*Give P values as exact values whenever suitable.* |
| ☒ | ☐ | For Bayesian analysis, information on the choice of priors and Markov chain Monte Carlo settings |
| ☒ | ☐ | For hierarchical and complex designs, identification of the appropriate level for tests and full reporting of outcomes |
| ☒ | ☐ | Estimates of effect sizes (e.g. Cohen's *d*, Pearson's *r*), indicating how they were calculated |

*Our web collection on statistics for biologists contains articles on many of the points above.*

## Software and code

Policy information about availability of computer code

| | |
|---|---|
| Data collection | All data and mathematical results of this work have been processed using MATLAB R2019b and Excel 2016. |
| Data analysis | All data and mathematical results of this work have been processed using MATLAB R2019b, Excel 2016 and Tableau Public 2019.4. The code for the computation of the GENEPY index is available at https://zenodo.org/record/3876721. |

For manuscripts utilizing custom algorithms or software that are central to the research but not yet described in published literature, software must be made available to editors/reviewers. We strongly encourage code deposition in a community repository (e.g. GitHub). See the Nature Research guidelines for submitting code & software for further information.

## Data

Policy information about availability of data

All manuscripts must include a data availability statement. This statement should provide the following information, where applicable:

- Accession codes, unique identifiers, or web links for publicly available datasets
- A list of figures that have associated raw data
- A description of any restrictions on data availability

The trade data supporting the findings of this study are available upon request from the BACI-CEPII database (ref.36). Downloads may require paid subscription.
The GDP PPP and population data used in this work are provided by The World Bank and publicly and freely available at https://data.worldbank.org/.
The data on the coordinates of countries are provided by the Portland State University and publicly and freely available at https://www.pdx.edu/econ/country-geography-data.
The pollinators-plants networks are freely available at www.web-of-life.es.
The results of the GENEPY index for countries during the period of analysis are publicly and freely available at https://zenodo.org/339 record/3876721. Other results are available from the authors upon request.

# Field-specific reporting

Please select the one below that is the best fit for your research. If you are not sure, read the appropriate sections before making your selection.

- [ ] Life sciences
- [x] Behavioural & social sciences
- [ ] Ecological, evolutionary & environmental sciences

For a reference copy of the document with all sections, see nature.com/documents/nr-reporting-summary-flat.pdf

# Behavioural & social sciences study design

All studies must disclose on these points even when the disclosure is negative.

| | |
|---|---|
| Study description | Quantitative. |
| Research sample | The trade data supporting the findings of this study are available upon paid request from the BACI-CEPII database (ref.36). |
| Sampling strategy | Not applicable. All published data have been used. |
| Data collection | Not applicable. Data are processed from the CEPII research group. |
| Timing | Yearly reported trade during 1995 - 2017. |
| Data exclusions | Our data include all the countries whose export share is worth at least 10^-5 of the total flux traded during the year (i.e., the total amount of dollars exported worldwide). For political reasons, Taiwan has been excluded from the analysis. |
| Non-participation | Not applicable. |
| Randomization | Not applicable. |

# Reporting for specific materials, systems and methods

We require information from authors about some types of materials, experimental systems and methods used in many studies. Here, indicate whether each material, system or method listed is relevant to your study. If you are not sure if a list item applies to your research, read the appropriate section before selecting a response.

## Materials & experimental systems

| n/a | Involved in the study | |
|---|---|---|
| x | | Antibodies |
| x | | Eukaryotic cell lines |
| x | | Palaeontology |
| x | | Animals and other organisms |
| x | | Human research participants |
| x | | Clinical data |

## Methods

| n/a | Involved in the study | |
|---|---|---|
| x | | ChIP-seq |
| x | | Flow cytometry |
| x | | MRI-based neuroimaging |
